# Women’s Autonomy and Respectful Maternity Care in Zambia: A Cross-Sectional Study Using Validated Patient-Reported Measures

**DOI:** 10.1007/s10995-026-04278-9

**Published:** 2026-05-09

**Authors:** Atika Khalaf, Francis Sichimba, Kalunga Cindy Nakazwe, Lena Halawi

**Affiliations:** 1 Faculty of Health Science, Kristianstad University, Elmetorpsvägen 15, 29188 Kristianstad, Sweden; 2 Hind Bint Maktoum College of Nursing and Midwifery, Dubai Health, Mohammed Bin Rashid University of Medicine and Health Sciences, Dubai, UAE; 3 Department of Psychology, University of Zambia, Lusaka, Zambia

**Keywords:** Women, Autonomy, Respectful care, Maternal healthcare services, MADM, MOR, Zambia

## Abstract

**Objective:**

Although women’s autonomy is key to patient care, the extent to which patients exercise autonomy during clinical consultations in resource-limited contexts such as Zambia remains unknown. This study aimed to examine women’s experiences of autonomy in decision-making and respectful treatment in Zambian maternal healthcare.

**Methods:**

The study was conducted in Lusaka and used a cross-sectional survey design. The sample consisted of 305 women who were conveniently recruited. Data was collected using a questionnaire consisting of sociodemographic data and two validated instruments: the 7-item Mothers Autonomy and Decision-Making Scale (MADM), and the 14-item Measure of Respect (MOR) index.

**Results:**

Higher levels of decision-making autonomy, according to MADM, are significantly associated with higher levels of perceived respect in maternal healthcare settings, according to MOR (*p* < .001). Furthermore, higher education was found to correlate significantly with higher autonomy and respect (*p* < .001). Both instruments showed an excellent (MADM) and good (MOR) internal consistency among this sample (Cronbach’s alpha = 0.959 and 0.851, respectively). Overall, while there’s significant variation in responses, there is a slight tendency towards positive experiences in both autonomy and respect in maternal healthcare settings.

**Conclusions for Practice:**

Health authorities should promote the implementation of autonomous and respectful care for all women, regardless of socioeconomic or educational background, and provide a supportive environment that fosters user participation in decision-making.

## Significance

What is Already Known on This Subject?

What Does This Study Add?

Patient autonomy is a core ethical principle in enhancing patient-centered care and influencing healthcare decision-making. However, the extent of maternal autonomy practice in resource-limited contexts like Zambia remains understudied.

This study reveals significant disparities in Zambian women’s experiences of autonomy and respectful care, influenced by sociodemographic factors and provider type – indicating potential systemic biases and structural inequities in Zambian maternity care. Higher education, employment, marital status, and having children correlate with increased autonomy and respect. Furthermore, women reported higher levels of autonomy and respect when consulting OB/GYNs than when consulting midwives, contrary to findings in other contexts.

## Introduction

Patient autonomy is now a widely accepted ethical principle in medical practice and significantly contributes to service quality in patient-centered care (Mapes et al., 2020). While limited cross-cultural studies exist, the autonomy model has profoundly influenced decision-making in healthcare (Racine et al., 2021), viewed as a moral imperative. Allowing patients to exercise autonomy (Greaney & Flaherty, 2020) enhances their respect as agents in health-related decisions (Molina-Mula & Gallo-Estrada, 2020). However, the extent of patient autonomy in clinical consultations, particularly in resource-limited contexts such as Zambia, remains unclear, especially in interactions with maternal healthcare services and providers.

In healthcare, patient autonomy refers to a patient’s right to make independent, rational decisions (Akhtar et al., 2024; Cave, 2020) about their care without undue influence from providers. The World Health Organization supports this principle, emphasizing the importance of patients’ rights to make health decisions (WHO, 2022). However, research on patient experiences of autonomy is limited. Autonomy can be hindered by patients’ dependent conditions and the traditional authority of healthcare professionals (Lindberg et al., 2014). Tensions often arise between patients’ choices and professional advice, as well as issues concerning accountability and policy versus practice (Greaney & O’Mathúna, 2024). Given the power dynamics in patient-provider interactions, understanding how clinicians can support patient autonomy is essential, which is the focus of this study.

Patients who experience autonomy in healthcare feel empowered and valued, gaining a sense of control over their health-related issues, which is linked to increased self-esteem and confidence. For example, research in Ethiopia showed that women’s autonomy positively influenced maternal healthcare utilization (Tiruneh et al., 2017). Making decisions about one’s own life, including healthcare choices, supports autonomy (WHO, 2022). However, cultural beliefs and the authority of doctors often hinder patients from exercising their autonomy, as they may feel prohibited from expressing their true preferences or questioning medical decisions (Akpa-Inyang & Chima, 2021). Further, a doctor’s authority and power can override the patient’s autonomous rights (Cave, 2020). Given these challenges and cultural barriers, it is important to study patients’ perspectives on autonomy in Zambia.

To foster autonomy, health providers must recognize patients as capable agents and understand that autonomy is a process (Frank et al., 2022) influenced by health status and the surrounding care environment (Pursio et al., 2021). Research shows that providers play a crucial role in promoting autonomy through patient-centered communication (Manalastas et al., 2020), enabling patients to voice their interests and make decisions (Knight, 2019). The extent to which healthcare providers support patients’ feelings of autonomy influences their ability to exercise it (Molina-Mula & Gallo-Estrada, 2020). Literature indicates that accepting patient opinions and encouraging participation in health decisions can enhance patient autonomy (Grünloh et al., 2018).

Zambia’s population growth affects healthcare services, but this increase doesn’t result in higher demands, leading to poor provider-patient ratios and shortages of health personnel (Mberu & Ezeh, 2017), particularly in rural areas. Maternal health is significantly impacted, with one in 160 women dying from pregnancy-related issues, compared to one in 3,700 in industrialized nations (Mbau et al., 2023). Common causes of maternal and perinatal fatalities include women’s failure to deliver in adequately resourced health facilities (Musonda et al., 2021). A study in Zambia indicated that nurses’ disrespectful attitudes and pregnant women’s negative perceptions of healthcare are significant barriers to accessing qualified care (Mukonka et al., 2024). Thus, exploring autonomy in maternal health services is crucial, as the care environment and providers’ moral values can influence practices (Sialubanje et al., 2023). The Health Professional Council of Zambia’s guidelines emphasize patient autonomy by requiring informed consent and addressing unethical practices like coercion (HPCZ, 2023). These standards promote voluntary decision-making in health services. However, the extent to which healthcare providers support autonomy in maternal health care remains unclear.

In Zambia, like in many traditional societies, medical professionals hold a privileged status because of the clinical knowledge they bring to the treatment of illness. Given the context characterized by high patient ratios, lack of specialized healthcare providers, and low literacy levels in rural areas, it makes for a suitable environment to study patient autonomy (Owino et al., 2025). Several barriers lead to low demand and limited access to maternity services, including cultural factors, provider attitudes, a lack of female providers, service quality, and insufficient awareness (Jacobs et al., 2023; Mweemba et al., 2021). However, significant gaps in patient autonomy in maternal services in Zambia remain a challenge. Thus, this study aimed to evaluate the quality of maternity care by examining women’s experiences of decision-making autonomy and respectful treatment. The findings sought to enhance understanding of patient autonomy in Zambian maternal health services and encourage adherence to ethical standards among healthcare providers.

## Methods

### Study Design

An exploratory-descriptive research design using a cross-sectional survey was employed, which incorporated a quantitative methodological approach. The research design was exploratory in that there appeared to be little evidence of formal research previously conducted on autonomy in maternal healthcare services (Elman et al., 2020). It was also descriptive, as the study sought to describe women’s knowledge and experiences with autonomy in maternal healthcare services.

### Sample Size

The sample size was estimated using the Cochran formula (Cochran, 1977): 𝑛=𝑍^2^⋅𝑝⋅(1−𝑝)/𝑒^2^, where 𝑍 was set to the typical value of 1.96, the estimated proportion (*p*) was set to 0.5 for maximum variability (when the proportion of the population that exhibits the characteristic of interest is unknown), and a margin of error (*e*) commonly set at 0.05. The equation led to a sample size of approximately 385 women. Given that Lusaka has a population of approximately 735,361 women of childbearing age, and with around 100,919 expected pregnancies annually (Hibusu et al., 2024), a sample size of 385 was considered statistically significant and should provide adequate power to detect differences in maternal autonomy across sociodemographic groups.

The link was shared with 500 women, and 305 ultimately responded to the online questionnaire. This achieved sample size was smaller than the initially calculated minimum of 385, which may have resulted in a slightly higher margin of error and reduced statistical precision than originally intended. The recruitment and data collection phases extended over six months, from May 2024 to October 2024.

### Setting

The research was conducted in Lusaka, a city of approximately 3,079,964 people (MoH, 2022). Despite better access to maternal health services than other regions in Zambia, challenges persist, including high morbidity and mortality rates (Mweemba et al., 2021). Many women do not receive timely or high-quality care (Jacobs et al., 2024), facing barriers such as concerns about service quality and provider attitudes. Thus, the study focused on women attending antenatal and postnatal services, highlighting Lusaka’s higher utilization of these services.

### Instruments

The survey included structured sociodemographic information, namely, age, educational level, employment status, marital status, number of children, and whether women had been in contact with maternal health services in the last two years.

Women’s experiences of autonomy in their interaction with maternal healthcare services during pregnancy, labor, and/or childbirth were assessed using a questionnaire consisting of 10 demographic questions, a 7-item “Mothers’ Autonomy in Decision Making Scale” (MADM), and a 14-item “Measure of Respect Index” (MOR).

#### Mothers’ Autonomy in Decision Making

MADM, a 7-item scale, is a validated instrument designed to assess maternal autonomy during maternity care. Developed through community engagement, the MADM scale comprises seven items that measure women’s ability to lead decision-making processes, the adequacy of time allotted to consider options, and the respect for their choices by healthcare providers. Responses are scored on a 6-point Likert scale from 1 (strongly disagree) to 6 (strongly agree), yielding a total score of 7–42, with higher scores indicating greater perceived autonomy.

Psychometric evaluations have demonstrated MADM’s strong reliability, with Cronbach’s alpha coefficients ranging from 0.93 to 0.97 across various populations, indicating excellent internal consistency (Vedam et al., 2017). The scale has been adapted and validated in different cultural contexts, including Dutch and Greek populations, confirming its applicability and effectiveness in measuring maternal autonomy in diverse settings (Peters et al., 2022; Serpetini et al., 2024).

#### Mothers on Respect Index

MOR is a 14-item self-report instrument designed to measure women’s experiences of respectful and disrespectful care from maternity care providers. This index assesses various dimensions of respectful care, including communication ease, patient autonomy, and perceptions of discrimination during maternity care. While the first seven items (MOR-A) are about the women’s overall feelings of respect while making decisions during their pregnancy, the following four items are about feelings of being treated poorly by the midwife or doctor after the mothers had their babies (MOR-B). The remaining three items are about experiences of holding back from asking questions or discussing the mothers’ concerns during a prenatal visit (MOR-C). Each item is scored on a 6-point Likert scale, ranging from 1 (strongly disagree) to 6 (strongly agree), resulting in a total score between 14 and 84, where higher scores indicate more respectful interactions with healthcare providers.

The MOR has demonstrated strong psychometric properties across diverse populations, including Canadian and Dutch women, with Cronbach’s alpha values ranging from 0.76 to 0.94, indicating good internal consistency (Vedam et al., 2017). The instrument has been validated for use in various cultural contexts, confirming its reliability as a quality and safety indicator in maternity care (Peters et al., 2022).

For the current study, the English versions of the MADM and MOR were utilized after obtaining permission from the instrument holder via email communication (BPL, n.d.).

### Data Collection

The research team, comprising two experienced researchers (the second and third authors) and a trained research assistant, implemented a systematic recruitment and data-collection process. The initial phase involved collaboration with maternal healthcare providers, predominantly nurses, at a major tertiary hospital in Lusaka. These healthcare professionals identified and recruited 50 women who met the predefined inclusion criteria and administered the validated questionnaire. To expand the sample, a snowball sampling technique was employed. The recruited women were provided with a comprehensive information sheet detailing the study’s purpose and the researchers’ contact information, which they were encouraged to share within their social networks, including church groups, WhatsApp communities, and workplaces. The research team then conducted a rigorous eligibility screening of potential participants who expressed interest. Those meeting the inclusion criteria were provided secure access to the online questionnaire, hosted on a GDPR-compliant platform (Google Forms). The questionnaire included a mandatory informed consent form that participants were required to complete before accessing the survey items, thereby ensuring ethical compliance and voluntary participation.

### Data Analysis

Data analysis was conducted using IBM SPSS Statistics version 27.0 (IBM Corp., Armonk, NY, USA). Descriptive statistics were used to summarize the participants’ sociodemographic characteristics, including means and standard deviations for continuous variables and frequencies and percentages for categorical variables.

Bivariate analyses were performed to examine the relationships between sociodemographic characteristics and the MADM and MOR scores. Pearson’s correlation coefficient was used for continuous variables, while independent t-tests or one-way ANOVA were employed for categorical variables, as appropriate. The strength of correlations was interpreted as weak (0.1–0.3), moderate (0.3–0.5), or strong (> 0.5).

To compare high and low levels of MADM and MOR scores across sociodemographic characteristics, the scores were dichotomised based on their distributions. Chi-square tests were then conducted, with Fisher’s Exact Test where applicable. Statistical significance was set at *p* < .05 for all analyses.

The distribution of responses to individual MADM and MOR items was analyzed using frequency tables. Additionally, the relationships between MADM and MOR items and their total scores were examined using Pearson’s correlation coefficient to identify associations between autonomy in decision-making and respect levels.

The internal consistency reliability of the MADM and the MOR was assessed using Cronbach’s alpha coefficient, with values ≥ 0.70 considered acceptable.

### Ethical Consideration

Ethical approval to conduct the study was obtained from the University of Zambia, Humanities and Social Sciences Research Ethics Committee (Ref. No. HSSREC-2023-OCT-028) on November 28, 2023. Participants were asked to sign consent forms before participating in the study. All data collection methods were implemented in accordance with the Helsinki Declaration.

## Results

The participating women (*n* = 305) were characterized as follows: their mean age was 31.3 years (SD = 8.8). The majority had a tertiary or higher level of education (59.7%), were employed full-time (69.2%), married (70.8%), had children (88.2%), and had been in contact with maternal healthcare services in the last two years (75.4%). The mean scores for the MADM and the MOR showed a statistically significant but weak correlation with participants’ sociodemographic characteristics (see Table 1).

**Table 1:** Mean scores of the Mothers Autonomy in Decision Making Scale (MADM) and the Mothers on Respect Index (MOR) based on participants’ sociodemographic characteristics

Characteristics	*n* (%)	MADM		MOR	
		m (SD)	Correlation	m (SD)	Correlation
Age, m (SD), min-max	31.3 (8.8), 15–68		0.160**		0.150**
*Education*			0.430**		0.341**
< Primary	47 (15.4)	13 (13)		44 (12)	
Primary to secondary	76 (24.9)	23 (14)		56 (16)	
Tertiary and higher	182 (59.7)	29 (11)		61 (15)	
*Employment*			0.130*		0.142*
Fulltime	211 (69.2)	26 (13)		58 (15)	
Parttime	19 (6.2)	19 (13)		52 (16)	
Jobseeker	65 (21.3)	23 (14)		54 (18)	
Other^#^	10 (3.3)	29 (14)		66 (16)	
*Marital status*			− 0.145*		− 0.123*
Single	56 (18.4)	25 (13)		58 (17)	
Married	216 (70.8)	26 (13)		59 (15)	
Widowed	9 (3.0)	20 (16)		45 (13)	
Divorced	15 (4.9)	20 (15)		48 (13)	
Separated	9 (3.0)	15 (13)		52 (13)	
*Have children*			− 0.174**		− 0.163**
Yes	269 (88.2)	26 (13)		58 (16)	
No^$^	17 (5.6)	22 (14)		50 (15)	
Expecting	19 (6.2)	17 (14)		50 (13)	
*Contact with MHC* ^^^			0.216**		**0.103**
Yes	230 (75.4)	27 (13)		58 (16)	
No	75 (24.6)	20 (13)		55 (13)	

**Correlation is significant at the 0.01 level (2-tailed)

*Correlation is significant at the 0.05 level (2-tailed)

^#^Other employment status includes being a full-time student

^$^Not having children includes having been pregnant but losing the baby (miscarriage, abortion)

^^^Been in contact with maternal healthcare (MHC) during the last 2 years

Bolded values indicate statistical significance

A comparison of high and low levels of the MADM and MOR scores across various sociodemographic characteristics is presented in Table 2. Education level was significantly associated with both MADM and MOR scores (*p* < .001), with higher education levels correlating with greater autonomy and respect. While the employment status was significantly associated with MADM scores (*p* = .017) but not with MOR scores (*p* = .216), the marital status was significantly associated with MOR scores (*p* = .033) but not with MADM scores (*p* = .178). Similarly, having children was associated significantly with MOR scores (*p* = .003) but only marginally with MADM scores (*p* = .068). Contact with MHC and the type of HCP both showed significant associations with both MADM and MOR scores (*p* < .001 for most comparisons). Notably, women who saw OB/GYNs reported higher levels of autonomy and respect compared to those who saw other types of providers.

**Table 2:** A group comparison between high and low levels of the Mothers Autonomy in Decision Making Scale (MADM) and the Mothers on Respect Index (MOR) based on participants’ sociodemographic characteristics. Data are presented in row percentage, n (%)

Characteristics	Sample, *n *(%)	MADM, *n*(%)		*p* value*	MOR		*p* value*
		High, *n* = 136	Low, *n* = 169		High, *n* = 150	Low, *n* = 155	
*Education*				< 0.001			< 0.001
< Primary	47 (15.4)	9 (19.1)	38 (80.9)		3 (6.4)	44 (93.6)	
Primary to secondary	76 (24.9)	38 (50.0)	38 (50.0)		37 (48.7)	39 (51.3)	
Tertiary and higher	182 (59.7)	122 (67.0)	60 (33.0)		110 (60.4)	72 (39.6)	
*Employment*				**0.017**			0.216
Fulltime	211 (69.2)	125 (59.2)	86 (40.8)		109 (51.7)	102 (48.3)	
Parttime	19 (6.2)	5 (26.3)	14 (73.7)		6 (31.6)	13 (68.4)	
Jobseeker	75 (24.6)	39 (52.0)	36 (48.0)		35 (46.7)	40 (53.3)	
*Marital status*				0.178			**0.033**
Single^^^	89 (29.2)	44 (49.4)	45 (50.6)		36 (40.4)	53 (59.6)	
Married	216 (70.8)	125 (57.9)	91 (42.1)		114 (52.8)	102 (47.2)	
*Have children*				0.068			**0.003**
Yes	269 (88.2)	155 (57.6)	114 (42.4)		142 (52.8)	127 (47.2)	
No^$^	36 (5.6)	8 (47.1)	9 (52.9)		4 (23.5)	13 (76.5)	
Expecting	19 (6.2)	6 (31.6)	13 (68.4)		4 (21.1)	15 (78.9)	
*Contact with MHC* ^**^**^				**< 0.001**			**0.013**
Yes	230 (75.4)	140 (60.9)	90 (39.1)		122 (53.0)	108 (47.0)	
No	75 (24.6)	29 (38.7)	46 (61.3)		28 (37.3)	47 (62.7)	
*Type of HCP*				**< 0.001**			**< 0.001**
Family doctor	16 (5.2)	9 (56.3)	7 (43.8)		6 (37.5)	10 (62.5)	
OB/GYN	114 (37.4)	83 (72.8)	31 (27.2)		77 (67.5)	37 (32.5)	
Midwife	147 (48.2)	73 (49.7)	74 (50.3)		65 (44.2)	82 (55.8)	
Other	28 (9.2)	4 (14.3)	24 (85.7)		2 (7.1)	26 (92.9)	

*HCP* Healthcare provider

*Pearson Chi-square (Fisher’s exact test where applicable)

^^^Single includes widowed, divorced, and separated women

Bolded values indicate statistical significance

For the MADM items (MADM-1 to MADM-7), there is a notable polarization in responses, with high percentages in both the “Strongly disagree” and “Strongly agree” categories, suggesting diverse experiences of autonomy among respondents. The MOR items show a similar pattern for positively worded questions (MOR-A1 to MOR-A7), with a tendency towards more positive responses. However, for the reverse-scored items (MOR-B1 to MOR-C3), there’s a strong skew towards “Strongly disagree,” which, when reversed, indicates generally positive experiences of respect. Notably, MOR-A4 (a positively worded item) has the highest percentage of “Strongly agree” responses at 52.5%, suggesting that most respondents felt their healthcare providers treated them with respect. Overall, while there’s significant variation in responses, there is a slight tendency towards positive experiences in both autonomy and respect in maternal healthcare settings. See Table 3 for further details.

**Table 3:** Frequency of the responses to the MADM and MOR items

Item	Responses, *n* (%)					
	Strongly disagree	Disagree	Somewhat disagree	Somewhat agree	Agree	Strongly agree
*MADM*						
MADM-1	128 (42.0)	17 (5.6)	23 (7.5)	19 (6.2)	36 (11.8)	82 (26.9)
MADM-2	111 (36.4)	18 (5.9)	17 (5.6)	27 (8.9)	35 (11.5)	97 (31.8)
MADM-3	101 (33.1)	12 (3.9)	23 (7.5)	24 (7.9)	38 (12.5)	107 (35.1)
MADM-4	92 (30.2)	13 (4.3)	20 (6.6)	21 (6.9)	36 (11.8)	123 (40.3)
MADM-5	104 (34.1)	18 (5.9)	20 (6.6)	21 (6.9)	45 (14.8)	97 (31.8)
MADM-6	106 (34.8)	14 (4.6)	17 (5.6)	25 (8.2)	35 (11.5)	108 (35.4)
MADM-7	91 (29.8)	15 (4.9)	19 (6.2)	26 (8.5)	37 (12.1)	117 (38.4)
*MOR*						
MOR-A1	77 (25.2)	9 (3.0)	23 (7.5)	26 (8.5)	37 (12.1)	133 (43.6)
MOR-A2	101 (33.1)	25 (8.2)	29 (9.5)	34 (11.1)	33 (10.8)	83 (27.2)
MOR-A3	74 (24.3)	12 (3.9)	32 (10.5)	28 (9.2)	48(15.7)	111 (36.4)
MOR-A4*	32 (10.5)	22 (7.2)	23 (7.5)	28 (9.2)	40 (13.1)	160 (52.5)
MOR-A5	80 (26.2)	16 (5.2)	32 (10.5)	32 (10.5)	40 (13.1)	105 (34.4)
MOR-A6	68 (22.3)	15 (4.9)	34 (11.1)	36 (11.8)	48 (15.7)	104 (34.1)
MOR-A7	70 (23.0)	7 (2.3)	36 (11.8)	38 (12.5)	48 (15.7)	106 (34.8)
MOR-B1*	189 (62.0)	30 (9.8)	19 (6.2)	22 (7.2)	12 (3.9)	33 (10.8)
MOR-B2*	191 (62.6)	21 (6.9)	18 (5.9)	25 (8.2)	11 (3.6)	39 (12.8)
MOR-B3*	178 (58.4)	35 (11.5)	20 (6.6)	23 (7.5)	14 (4.6)	35 (11.5)
MOR-B4*	154 (50.5)	35 (11.5)	23 (7.5)	39 (12.8)	15 (4.9)	39 (12.8)
MOR-C1*	139 (45.6)	29 (9.5)	17 (5.6)	39 (12.8)	19 (6.2)	62 (20.3)
MOR-C2*	147 (48.2)	35 (11.5)	23 (7.5)	34 (11.1)	15 (4.9)	51 (16.7)
MOR-C3*	137 (44.9)	33 (10.8)	20 (6.6)	39 (12.8)	18 (5.9)	58 (19.0)

*MADM* Mother’s Autonomy in Decision Making scale, *MOR* Mothers on Respect index

*Reverse-scored items

Table 4 shows a strong positive relationship between women’s autonomy in decision-making and their experiences of respect in maternal healthcare. Most MOR-A items show strong positive correlations with all MADM items, while MOR-B items show little to no correlation, and MOR-C items demonstrate weak to moderate positive correlations. Notably, the MADM total score has a strong positive correlation with both the MOR-A score (*r* = .831) and the overall MOR total score (*r* = .671). However, MOR-A4 stands out with weak negative or near-zero correlations with MADM items, suggesting this particular aspect of respect is not related to decision-making autonomy. The findings indicate that greater autonomy in decision-making is associated with higher perceived respect in maternal healthcare settings, particularly for aspects measured by the MOR-A subscale.

**Table 4:** The relationship between MADM and MOR items and total scores

MOR Items	MADM Items							MADM total score
	MADM-1	MADM-2	MADM-3	MADM-4	MADM-5	MADM-6	MADM − 7	
MOR-A1	0.549**	0.657**	0.699**	0.750**	0.667**	0.682**	0.726**	
MOR-A2	0.500**	0.515**	0.520**	0.604**	0.546**	0.587**	0.572**	
MOR-A3	0.588**	0.676**	0.697**	0.752**	0.699**	0.752**	0.757**	
MOR-A4	0.029	0.021	− 0.040	− 0.058	− 0.021	− 0.051	− 0.093	
MOR-A5	0.576**	0.620**	0.628**	0.675**	0.692**	0.719**	0.791**	
MOR-A6	0.561**	0.616**	0.632**	0.700**	0.683**	0.724**	0.789**	
MOR-A7	0.500**	0.622**	0.623**	0.680**	0.651**	0.663**	0.718**	
*MOR-A score*								0.831**
MOR-B1	0.047	0.071	0.072	0.093	0.058	0.072	0.041	
MOR-B2	− 0.054	− 0.012	0.023	0.051	− 0.020	0.011	− 0.011	
MOR-B3	0.017	0.045	0.028	0.081	0.051	0.064	0.068	
MOR-B4	− 0.004	0.059	0.014	0.044	0.042	0.074	0.086	
*MOR-B score*								0.028
MOR-C1	0.252	0.240**	0.213**	0.298**	0.227**	0.275**	0.276**	
MOR-C2	0.146	0.147**	0.128**	0.190**	0.135**	0.184**	0.203**	
MOR-C3	0.231	0.180**	0.168**	0.275**	0.182**	0.244**	0.255**	
MOR-C score								0.287**
MOR total score								0.671**

**Correlation is significant at the 0.01 level (2-tailed)

A reliability test of MADM and MOR among our sample showed a Cronbach’s Alpha of 0.959 and 0.851, respectively, indicating excellent internal consistency for the MADM scale and good internal consistency for the MOR scale. These values suggest that the instruments are reliable measures of their respective constructs, with the MADM demonstrating a very high level of reliability (above 0.95) and the MOR achieving a satisfactory level (above 0.80).

## Discussion

The study’s main findings reveal a complex interplay of sociodemographic factors influencing women’s experiences of autonomy and respectful care in Zambian maternity settings. Higher educational attainment, particularly tertiary education, strongly correlates with increased decision-making autonomy and respectful care. Similarly, employment status emerged as a significant predictor of women’s autonomy, with employed women reporting more active participation in healthcare decisions. Furthermore, both marital status and having children were linked to perceptions of respectful care. Notably, women who consulted OB/GYNs reported higher levels of autonomy and respect than those who received care from midwives. In addition to these associations, the overall mean scores of the MADM and MOR provide important insight into the general level of autonomy and respectful care experienced by women in Lusaka. While the findings indicate a slight tendency toward positive experiences, the variability and polarization in responses suggest that autonomy and respectful care are not uniformly experienced across the population. This pattern points to underlying systemic and structural inequalities, where certain groups of women may benefit from more supportive care environments than others. These disparities may reflect differences in health literacy, provider attitudes, and access to resources, reinforcing the need to address inequities in maternal healthcare delivery. Thus, the interpretation of mean scores should not only reflect central tendencies but also account for distributional differences that signal unequal experiences across sociodemographic groups.

This study demonstrates a significant and consistent association between women’s autonomy in decision-making and their experiences of respectful maternity care in Lusaka, Zambia, with greater autonomy closely associated with greater perceived respect. These findings suggest that autonomy and respectful care are co-constructed within clinical encounters, where the quality of provider-patient interaction plays a central role. Women who perceive respectful communication, empathy, and inclusion are more likely to actively participate in decision-making processes, reinforcing autonomy as a relational and context-dependent construct. This is consistent with existing evidence indicating that respectful, patient-centered communication fosters shared decision-making and enhances patients’ sense of agency (Manalastas et al., 2020; Molina-Mula & Gallo-Estrada, 2020). Furthermore, studies on person-centered maternity care have shown that respectful treatment and autonomy are deeply interconnected dimensions of quality care, often shaped by provider attitudes and health system factors (Afulani et al., 2019; Vedam et al., 2017). Collectively, these findings underscore the importance of strengthening interpersonal aspects of care, particularly communication, dignity, and mutual respect, as key mechanisms for improving both autonomy and overall maternal healthcare experiences.

Our findings highlight a strong connection between educational attainment and women’s autonomy in maternal decision-making, with tertiary education as a crucial threshold for improved outcomes. Women with low educational attainment showed significantly less autonomy and lower respect in care, aligning with global evidence connecting education to health agency (Rizkianti et al., 2020; Sougou et al., 2020). This underscores education’s dual role in fostering cognitive and social empowerment, consistent with the health capability framework (Acharya et al., 2010). Notably, the importance of tertiary education in Zambia, compared to other LMICs where primary education shows benefits (Haque et al., 2012; Nigatu et al., 2014), may reflect unique systemic barriers within Zambia’s healthcare context, such as provider biases towards educated patients. Importantly, these differences should be interpreted as evidence of underlying health inequalities. Women with lower educational attainment appear to experience reduced autonomy and respect, which may reflect structural disadvantages, including limited health literacy, reduced confidence in engaging with healthcare providers, and potential provider bias. Similarly, disparities observed across employment and marital status groups suggest that socially and economically disadvantaged women may be particularly vulnerable to suboptimal maternal care experiences. These findings underscore the need to view sociodemographic differences not merely as statistical variations but as indicators of inequitable care that require targeted policy and practice interventions. Further, this finding suggests that healthcare providers may benefit from fostering an autonomy-supportive climate, particularly when working with women who have low levels of formal education.

While the study confirms that primary education enhances autonomy compared to no formal schooling (Nigatu et al., 2014), it also highlights significant disparities across educational levels, indicating structural inequities in care delivery. Providers may unconsciously associate advanced education with competence (Afulani et al., 2021), leading to more participatory consultations for educated women, whereas less educated women may experience paternalistic care. This issue is worsened in rural Zambia due to limited health infrastructure and workforce shortages (Nankamba & Mwanaumo, 2024). Additionally, educated women may possess better cognitive and social skills, aiding their healthcare navigation (Acharya et al., 2010). These findings call for policy interventions to address provider biases and structural barriers to ensure equitable care, rather than relying solely on educational attainment.

Moreover, previous studies have highlighted disparities in respectful care linked to women’s socioeconomic status (Afulani et al., 2019). Investigations, particularly in Kenya (Afulani et al., 2021), reveal that maternity healthcare providers may hold biases based on women’s health literacy. Educated women are often perceived as more capable, leading to more respectful treatment and greater autonomy in decision-making (Afulani et al., 2021). Conversely, those with low levels of education may be viewed as less informed. This dynamic may clarify the connection between higher education and elevated MADM and MOR scores, suggesting that providers may offer better care to women they believe have higher expectations and are more adept at self-advocacy. Furthermore, employment status emerged as a significant factor related to the level of decision-making autonomy among women. This finding is consistent with prior research indicating that employment status can greatly affect decision-making autonomy, with women in paid employment more likely to report active participation in final decision-making than women not employed (Acharya et al., 2010). Employed women tend to play a more active role in decision-making, likely due to the positive effects of employment on their sense of self-reliance, which enables them to engage more fully in the decision-making process (Honjo et al., 2020). Additionally, marital status was significantly associated with higher level of respectful care, compared to women not in a marital union, which may be due to the result of companionship, which is indicative of healthcare providers, most likely, practicing caution in the way that they act and speak to the married women in the presence of the spouse, which is fortified by previous studies on the matter (Amsalu et al., 2022). In addition, having children was associated with higher MOR scores and higher levels of respectful care. This suggests a connection between experiencing multiple births and receiving respectful care, which aligns with findings from studies in India (Folchman-Wagner, 2025) and Ethiopia (Amsalu et al., 2022). It can be posited that previous childbirth experiences significantly influence this relationship due to a sense of familiarity with the process and expectations, which may enhance interactions with healthcare providers, resulting in an increased likelihood of receiving care that is respectful (Amsalu et al., 2022). Concerningly, our results showed that women consulting OB/GYNs experienced greater autonomy and respect than those cared for by midwives. This contrasts with other contexts in which midwifery-led care is associated with better patient experiences (Niles et al., 2023). In Zambia, this disparity may stem from challenges within the midwifery workforce, such as perceived competency gaps and trust issues (Nankamba & Mwanaumo, 2024; O’Brien et al., 2021). Further research is needed to understand these patient perceptions and improve midwifery care.

The findings underline the necessity for multifaceted interventions to ensure equitable maternity care in Zambia. Addressing implicit biases among healthcare providers, particularly related to socioeconomic status, is crucial (Afulani et al., 2021). These findings carry important theoretical and policy implications. The strong interrelationship between autonomy and respectful care reinforces patient-centered care models that conceptualize autonomy as relational and embedded within provider-patient interactions. From a policy perspective, the observed disparities across sociodemographic groups highlight the need for targeted interventions to address inequities in maternal healthcare. Key strategies include structured training for healthcare providers on respectful communication, shared decision-making, and implicit bias reduction, alongside fostering a culture of respect for all women. At the same time, structural interventions, such as improving women’s education, expanding employment opportunities, and strengthening community-level support, are essential to address systemic inequities and empower women to actively participate in healthcare decisions. Additionally, tailored approaches are needed to support vulnerable groups, particularly women with low levels of education and socioeconomic status. Embedding respectful maternity care standards into national guidelines and monitoring frameworks may further ensure equitable, high-quality care delivery across settings.

Future research should build on these findings by employing more rigorous sampling strategies, such as probability-based sampling, to enhance representativeness and generalizability. Qualitative studies are also warranted to explore in greater depth why women perceive varying levels of autonomy and respect, particularly among vulnerable or underrepresented groups. Additionally, longitudinal research designs would be valuable in examining how autonomy and respectful care evolve over time and in identifying potential causal relationships between sociodemographic factors and maternal healthcare experiences. Such approaches would provide a more comprehensive understanding of the mechanisms underlying autonomy and respectful care in maternal health settings.

### Strengths and Limitations

One notable limitation is the reliance on convenience sampling combined with an online recruitment approach, which introduces a risk of selection bias. The sample is likely skewed toward women with internet access, digital devices, and higher literacy levels, potentially excluding those from lower socioeconomic backgrounds or with limited technological access. As a result, certain vulnerable or marginalized groups may be underrepresented. Consequently, the findings cannot be strictly generalized to all women receiving maternal healthcare services in Lusaka and should be interpreted with caution. Furthermore, the cross-sectional design of this study precludes causal inferences; the findings should be interpreted as associations between sociodemographic characteristics and MADM and MOR scores rather than as evidence of cause-and-effect relationships. Additionally, there is the risk of recall bias often found in self-report surveys. Nonetheless, past research indicates that women can reliably recall experiences related to pregnancy, childbirth, and breastfeeding, even several years later (Amissah et al., 2017). Despite these limitations, this study has significant strengths, particularly its quantitative approach to evaluating maternity care quality from a sizable sample of women and the verification that all participants met the eligibility criteria for the study. Nevertheless, this research represents a positive step towards generating interest and expanding the literature on autonomous and respectful care in Zambia.

### Conclusions for Practice

In Zambia, disparities in maternal care and decision-making reveal systemic inequities in healthcare access among different sociodemographic groups. To address these issues, it is essential to implement policy reforms that prioritize women’s autonomy and dignity, particularly for underprivileged populations. Policymakers should establish evidence-based frameworks and national guidelines for standardized training of healthcare providers, especially midwives, and promote culturally sensitive care. Empowering women through active participation in decision-making and enhancing healthcare workers’ skills to counter biases are crucial strategies. Additionally, community education initiatives are vital for improving health literacy and empowering women to advocate for their rights, ensuring Zambia’s maternal healthcare system promotes dignity, equity, and justice for all women.

## Data Availability

The datasets generated and/or analysed during the current study are available from the first author on reasonable request.

## Code Availability

NA.
